# Curcumin: a natural organic component that plays a multi-faceted role in ovarian cancer

**DOI:** 10.1186/s13048-023-01120-6

**Published:** 2023-03-01

**Authors:** Xiaoping Liu, Mingming Qi, Xidie Li, Jingjin Wang, Mingyuan Wang

**Affiliations:** 1 Department of gynaecology and obstetrics, the Affiliated Zhuzhou Hospital Xiangya Medical College, Central South University, 412000 Zhuzhou, Hunan China; 2 National Clinical Research Center for Geriatric Disorders, Xiangya Hospital, Central South University, 410008 Changsha, Hunan China; 3 Department of Geriatric Surgery, Xiangya Hospital, Central South University, 410008 Changsha, Hunan China

**Keywords:** Curcumin, Ovarian cancer, Anti-tumor, Molecular mechanism, Review

## Abstract

Curcumin, a natural organic component obtained from Curcuma longa’s rhizomes, shows abundant anti-tumor, antioxidant and anti-inflammatory pharmacological activities, among others. Notably the anti-tumor activity has aroused widespread attention from scholars worldwide. Numerous studies have reported that curcumin can delay ovarian cancer (OC), increase its sensitivity to chemotherapy, and reduce chemotherapy drugs’ side effects. It has been shown considerable anticancer potential by promoting cell apoptosis, suppressing cell cycle progression, inducing autophagy, inhibiting tumor metastasis, and regulating enzyme activity. With an in-depth study of curcumin’s anti-OC mechanism, its clinical application will have broader prospects. This review summarizes the latest studies on curcumin’s anti-OC activities, and discusses the specific mechanism, hoping to provide references for further research and applications.

## Introduction

Curcumin is a natural hydrophobic polyphenol compound isolated from the rhizome of *Curcuma longa* (Turmeric) [1]. The chemical formula of curcumin is C_21_H_20_O_6_ and the relative molecular mass is 368.39. Curcumin consist of three main bioactive components, curcuminoid (70%), bisdemethoxycurcumin (10–20%) and demethoxycurcumin (10%). Among other roles, this natural polyphenolic compound acts as an antioxidant [2, 3], anti-aging [4], anti-inflammatory [5, 6], lipid-modifying [7, 8]. As a broad-spectrum anticancer drug, has been reported to selectively kill cancer cells through various biological pathways without toxic side effects on normal cells [9, 10]. These biological pathways include the induction of apoptosis [11, 12], cell cycle arrest [13–15], effects on autophagy [12, 16, 17], inhibition of tumor cell metastasis [18, 19], regulation of enzyme activity [20] and inhibition of the inflammatory response. Clinical trials have shown that curcumin does not have toxic and side effects at a dose of 8 g per day, which indicates its safety [21]. At present, a series of curcumin derivatives have been designed by modifying its chemical structure to make up for the limitations of its clinical application.

**Fig. 1:**
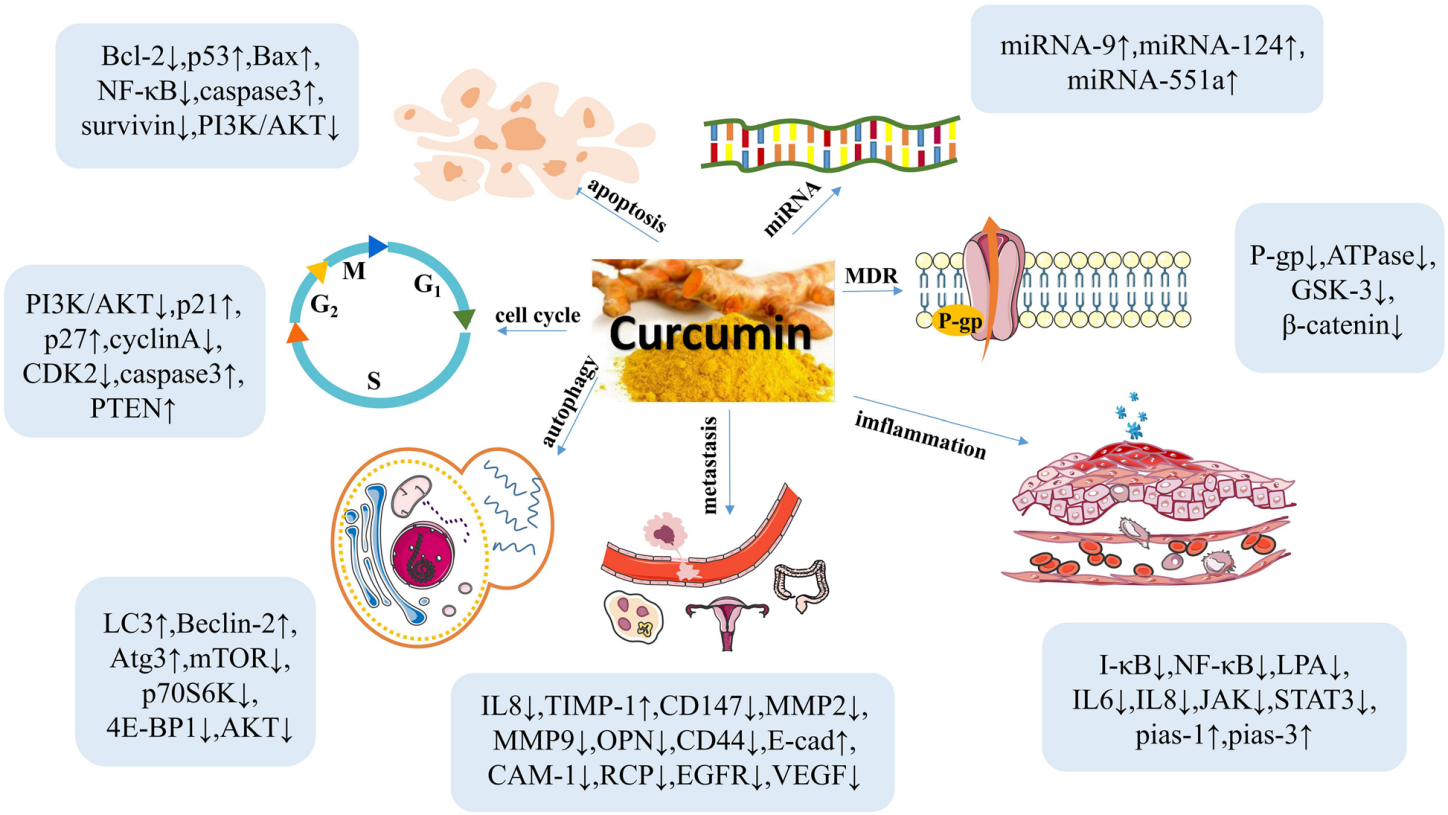
The main biological effects of curcumin on OC and the molecular targets involved

Ovarian cancer (OC) is one of the most common cancer worldwide and the fifth leading cause of cancer-related deaths in women [22, 23]. The current treatment is mainly surgical resection combined with postoperative chemotherapy and other comprehensive treatments. However, the side effects of chemotherapy drugs and susceptibility to drug resistance have become one of the main reasons for treatment failure [24]. Therefore, it is an urgent to discover new therapies with reduced toxicity and improved efficacy. Given its known advantages, including low toxicity, curcumin is expected to become an important auxiliary therapeutic agent. This review discusses the current research progress of curcumin’s anti-OC mechanisms (Fig. 1), providing references for further research and applications.

### Effects of curcumin on apoptosis

Apoptosis is closely associated with tumorigenesis, development, and metastasis of tumors. Typically, cancer cells escape from apoptosis. Several studies have reported that curcumin can mediate tumor cell apoptosis through modulating apoptosis-related proteins and activating signaling pathways. One of the mechanisms that induce apoptosis may be through down-regulating the expression of BCL-2 [25]. Compared with normal ovarian tissues, the anti-apoptotic protein BCL-2 is highly expressed in the OC epithelium [26]. The experimental results revealed that curcumin preconditioning downregulated expression of the anti-apoptotic protein Bcl-2, Bcl-X(L) and pro-caspase-3, while upregulated the pro-apoptotic protein molecule p53 and Bax. Curcumin could significantly suppressed the growth as well as induced the apoptosis of Ho-8910 cells [27]. Wang et al. [28]found the same results in SKOV3 cells. With an increase in curcumin concentration, the ratio of p-AKT to AKT decreased dose-dependently, and cytotoxicity increased markedly. This result suggested that curcumin might be an important inhibitor of the PI3K/AKT-signaling pathway [29]. In A2780 cells, curcumin time-dependently up-regulates caspase-3 to inhibit tumor growth [30]. Zhao et al [31] found that the activity of BCL-2 in SKOV3 cells was obviously decreased after curcumin treatment. Surprisingly, a marker of late apoptosis Caspase-3 remains unchanged, suggesting that curcumin may induce SKOV3 cells apoptosis in a manner independent of the Caspase-3 pathway. These results related subtle differences in the pathway of curcumin-induced apoptosis in different OC cell lines.

Overexpression of the anti-apoptotic protein Survivin is often associated with advanced tumors, poor prognosis, and tumor aggressiveness [32, 33]. Curcumin induces apoptosis of OC cells by downregulating the expression of Survivin and BCL-2 in SKOV3 cells and simultaneously activating P38 mitogen-activated protein kinase (MAPK), and this apoptosis induction effect is independent of p53 [34]. Interestingly, curcumin strongly activated adenylate-activated protein kinase (AMPK) in a p38-dependent manner and induced p53 phosphorylation in CaOV3 cells. Curcumin-induced cell death was reduced and p53 phosphorylation was inhibited after pretreated with AMPK and p38 inhibitors in CaOV3 cells [35]. Curcumin is also combined with the tumor necrosis factor-related apoptosis-inducing ligand (Apo2L / TRAIL) to enhance apoptosis. This combination activates the mitochondrial and death receptor pathway of apoptosis to evade resistance to conventional chemotherapy drugs [36].

Curcumin derivatives showed apoptosis induction in OC. Cleaved(activated) caspase-9 increased after the exposure of OC cells to the novel compound derivatives ST03 and ST08, which activates the mitochondrial intrinsic apoptosis pathway of OC to induce cytotoxicity [37]. B19, a novel monocarbonyl analog of curcumin, also induced apoptosis in OC [38] and activated caspase-3 in a time- and dose-dependent manner [39].

The endoplasmic reticulum (ER) is a multifunctional organelle which plays a vital role in protein folding and lipid biosynthesis. ER homeostasis may be destroyed in many physiological and pathological conditions. This will interfere with normal protein folding, resulting in the cumulation of unfolded or misfolded proteins in the lumen of the ER, conclusively inducing ER stress [40]. ER stress also triggers the unfolded protein response (UPR). When dysfunctional UPR cannot correct ER stress, the cell death program is activated [41]. A variety of anticancer agents can stimulate the UPR signaling in cancer cells. Thus, the possibility of B19-induced apoptosis through ER stress has been addressed. B19 can promote the accumulation of ubiquitinated misfolded proteins and trigger ER stress to induce OC cell apoptosis [38]. It also dose-dependently induced the expression of the downstream transcription factors XBP-1, ATF-4, and CHOP of UPR [39].

**Fig. 2:**
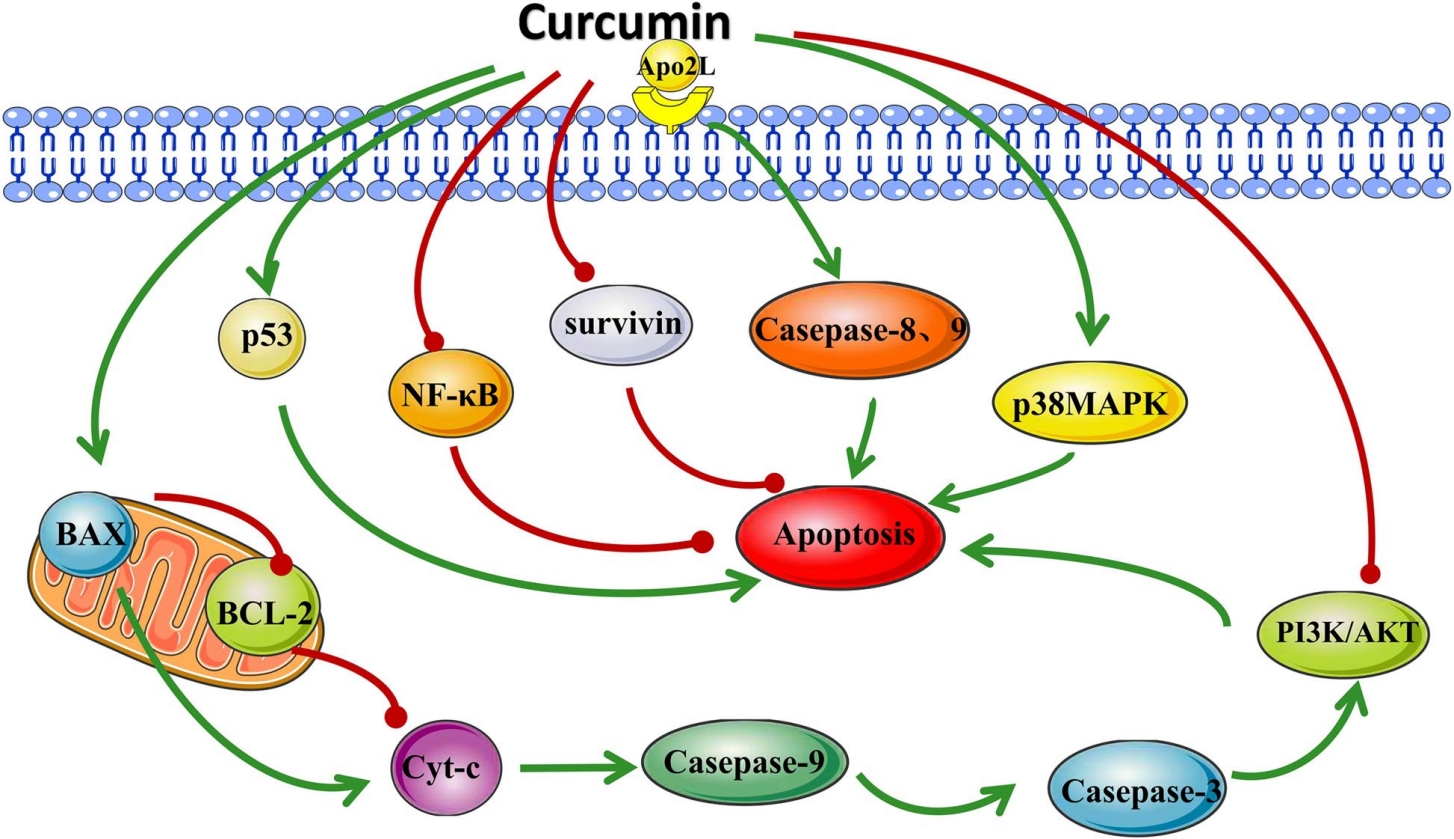
Curcumin’s molecular targets for apoptosis induction in OC cells. The green line indicates upregulation, while the red line indicates the downregulation of molecular targets. BCL-2: B-cell lymphoma-2; BAX: BCL-2-associated X protein; Cyt-c: cytochrome c; p53: Tumor protein 53; NF-κB: nuclear factor kappa B; Survivin: anti-apoptosis protein; caspase-3, 8, 9: cysteinyl aspartate-specific proteinase-3, 8, 9; p38 MAPK: p38 mitogen-activated protein kinases; PI3K: phosphatidylinositol 3-kinase; AKT: protein kinase

As summarized in Fig. 2, curcumin induces apoptosis through the modulation of numerous molecular targets. Thus, targeting the molecular pathways of apoptosis might be an effective approach in OC therapy.

### Effects of curcumin on cell cycle

Gene ontology (GO) enrichment analysis revealed many typical biological processes related to gynecological cancer. The consequences of this analysis indicate that the frequent biological processes of OC are those implicated in the regulation of cell cycle and macromolecular metabolism [42]. Generally, cells follow the G_0_/G_1_-S-G_2_-M cycle progression sequence. Curcumin can block the cell cycle of OC cells at different stages, thus inhibiting their disordered proliferation [43, 44]. SKOV-3 cells were treated with curcumin resulted in decreased cell populations in G1/G0, S, and G2/M phases [45]. A study by Yu et al [29] showed that curcumin arrested cell cycle progression in G2/M phase by down-regulating the PI3K/AKT pathway, thereby increasing the expression of caspase-3 and BAX. Furthermore, it can significantly reduce the level of BCL-2 and synergistically induce apoptosis. When curcumin and triptolide are used together in OC, they blocked the cell cycle at the S-phase and G2/M transition, exhibiting a powerful ability to induce apoptosis [46]. Nathan et al [44] further pointed out that curcumin elevates the phosphorylation of p53 by activating caspase-3 and PARP degradation, thereby leading to cell apoptosis and cell cycle arrest in G2/M phase.

Prior studies have suggested that the G2/M phase of the cell is regulated by several CDK/cyclins and CDK inhibitors (such as p21 and p27) [47]. HO3867, a new curcumin analog, could regulate the expression of cell cycle regulation molecules(p53, p21 and p27), cyclin-dependent kinase 2 and cyclin, activate Caspase-8 and Caspase-3 simultaneously. This causes the G2/M cell cycle of A2780 cells to arrest and eventually leads to cell apoptosis [48]. HO3867 also plays the same role in OC cisplatin-resistant cells (CR). When combined with cisplatin, HO3867 can enhance the sensitivity of CR cells, which may also be related to the upregulation of cell cycle regulatory proteins (such as p53, p21 and p27) thereby inducing G_2_/M phase arrest [49]. EF24, another compound with a structure similar to curcumin, also exhibits tumor-suppressive effects in various malignant tumors. It greatly inhibits the proliferation of CR cells through the induction of G_2_/M arrest and apoptosis. The cytotoxic effect of EF24 is also related to its inhibition of the degradation of pPTEN, the protein encoded by the *PTEN* tumor suppressor gene [47]. On the whole, these above findings indicate that curcumin inhibits disordered proliferation of tumor cells by inducing cell cycle arrest in G 2/M phase via different pathways, such as regulation of cell cycle-related proteins, apoptosis-related proteins.

### Effects of curcumin on autophagy

The anti-tumor activity of curcumin has been confirmed in various cancers, but some studies have pointed out that curcumin-induced autophagy prolongs the survival time of cancer cells, which may confuse the application of its anti-cancer properties [50]. Autophagy has a dual role in the tumor occurrence and development. In the early stage of tumor formation, autophagy degrades damaged organelles to repair mutated cells. In this process, autophagy plays a tumor-suppressing role. Under the condition of stress such as starvation and hypoxia, autophagy allows tumor cells that lack nutrition to degrade certain substances of their own to meet survival needs. This is an active adaptive response to stress, namely, protective autophagy. How curcumin affects autophagy in tumor cells is not very clear. In different types of malignant diseases, curcumin regulates autophagy differently [51–53]. Liu et al [12] found that curcumin significantly induce LC3, ATG3, and Beclin1 expression in a dose-dependent manner, and increase protective autophagy in OC cells. And further clarify that the underlying mechanism of this effect is through inhibiting the AKT/mTOR/p70S6K signaling pathway. After exposure to curcumin, the autophagic flux of OC cells increased in a dosage-dependent manner. Curcumin combined with the autophagy inhibitor chloroquine (CQ) or the knockdown of LC3B by siRNA significantly enhanced the sensitivity of cells toward curcumin treatment. These results revealed that autophagy induced by curcumin protects cancer cells from death. Simultaneously, Qu et al [38] found that curcumin’s new monocarbonyl analog B19 can induce OC cells death by apoptosis and autophagy. Apoptosis significantly increased after combined treatment with the curcumin and autophagy inhibitor 3-MA. This indicated that autophagy can help HO 8910 cells prevent the cell death induced by B19. Therefore, the combination of autophagy inhibitors and curcumin is expected to solve the resistance of curcumin to OC.

### Effects of curcumin on metastasis

**Fig. 3:**
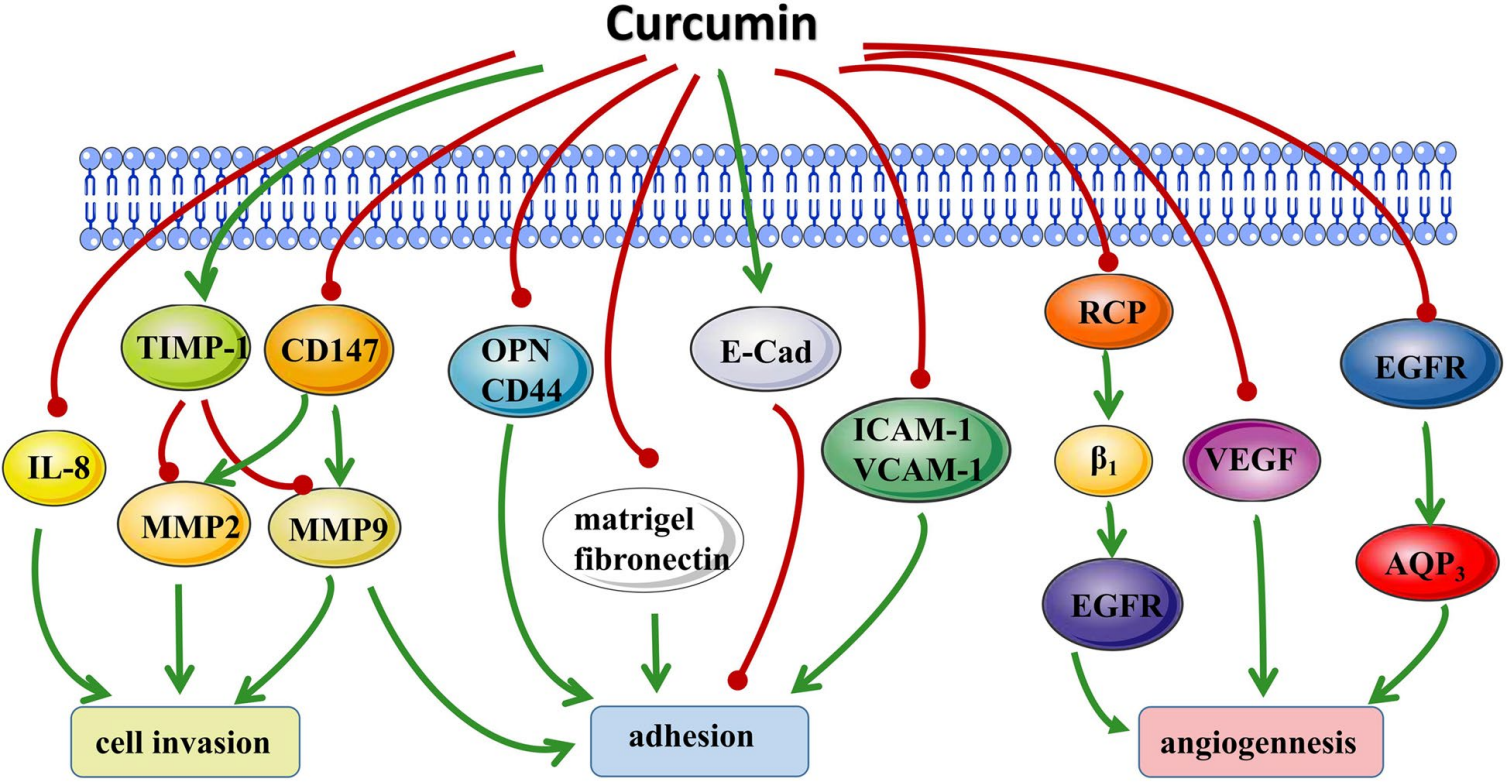
Molecular targets of curcumin in deregulating cell metastasis of OC cells. IL-8: Interleukin-8; TIMP-1: tissue inhibitor of metalloproteinases 1; CD147: extracellular matrix metalloproteinase inducer; MMP-2, -9: matrix metalloproteinase-2, -9; OPN: osteopontin; CD44: Cell surface antigen 44; E-cad: E-Cadherin; CAM: Calmodulin; RCP: Receptor component protein; EGFR: epidermal growth factor receptor; VEGF: vascular endothelial growth factor; AQP3: aquaporin water channel 3

Curcumin can inhibit tumor metastasis. Tumor metastasis includes cell invasion, adhesion, and angiogenesis. It has been shown that curcumin inhibits metastasis in OC cells by modulating several molecular targets including matrix metalloproteinases (MMPs), E-Cadherin (E-Cad) and vascular endothelial growth factor (VEGF; Fig. 3). It is a vital step that proteolytic enzyme-induced degradation of extracellular matrix (ECM). The two primary enzymes that degrade ECM elements include matrix metalloproteinase (MMP) and urokinase plasminogen activator (uPA). Curcumin can reduce the expression and activity of MMP and uPA through different ways, and is potentially used to inhibit cancer metastasis. Bisdemethoxycurcumin (BDMC, curcumin’s main active ingredient) obviously decrease mRNA and protein expression of MMP-2, MMP-9, and uPA in vitro studies as well. As is well known that TIMP-1 is considered as a negative regulator of MMP 2/9, while CD147 is considered as a positive regulator. As intended, BDMC obviously enhanced the content of TIMP-1 and decreased the level of CD147 protein. Research also confirms that MMP secretion may be affected by oxidative stress [54]. In vivo studies found that curcumin lowered the expression of MMP-9 [55], VEGF, and IL-8, and inhibited OC invasion [56]. Together, these results indicate that curcumin exerts anti-cancer effects by regulating the extracellular matrix degradation-related proteins.

Cell adhesion factor (CAM) mediates contact between cells and ECM, OC spread is associated with a loss of function of CAM such as E-Cad [57]. E-Cad proved to be an invasion and metastasis inhibitor in various human tumor tissues [58]. Curcumin attenuates lncRNA H19-induced epithelial-mesenchymal transition by the increase of N-Cad and the decrease of E-cad [59].After curcumin treatment, the E-Cad in the tumor was upregulated, the expression of interstitial markers such as fibronectin and vimentin decreased, and the cell migration ability decreased [60]. Curcumin can block the synthesis and release of CAM, thereby further inhibiting the formation of new blood vessels [54]. Curcumin inhibits the metastasis of OC cells and is also related to the reduction of osteopontin (OPN), CD44 and MMP-9 [55]. OPN can promote cell adhesion and invasion, both during physiological and pathological conditions [61]. These roles are primarily achieved through interacting with cell surface receptors (such as CD44) and activating downstream signaling pathways [62, 63].

The growth and metastasis of tumors largely depend on tumor angiogenesis. Tumor cells obtain nutrients through the process of tumor angiogenesis, which feeds uncontrolled growth. Curcumin inhibits pro-angiogenic factors (such as the expression of VEGF) to block the VEGF-VEGFR2 signaling pathway to inhibit VEGF-induced tumor proliferation and migration [64]. Curcumin also targets the NF-κB pathway to inhibit angiogenesis in OC [56]. In addition, the results of Ji et al. provide evidence that aquaporin (AQP3) promotes OC migration, and curcumin attenuates the epidermal growth factor receptor (EGFR) -induced AQP3 upregulation and cell migration in OC cells [65]. For the first time, Choe et al [66] demonstrated that curcumin inhibits the activation of EGFR by blocking the stabilizing effect of β1 integrin induced by Rab-coupled protein (RCP), thereby effectively inhibiting cell invasion in OC. Curcumin derivative WZ10 can significantly inhibit the proliferation of OVCAR3 cells, reduce cell invasion and proliferation by downregulating the activation of Hippo-YAP pathway, and induce cell apoptosis [67]. In short, curcumin regulates most of the classical pathways related to tumor metastasis to play an anti-cancer role.

### Effects of curcumin on inflammation

**Fig. 4:**
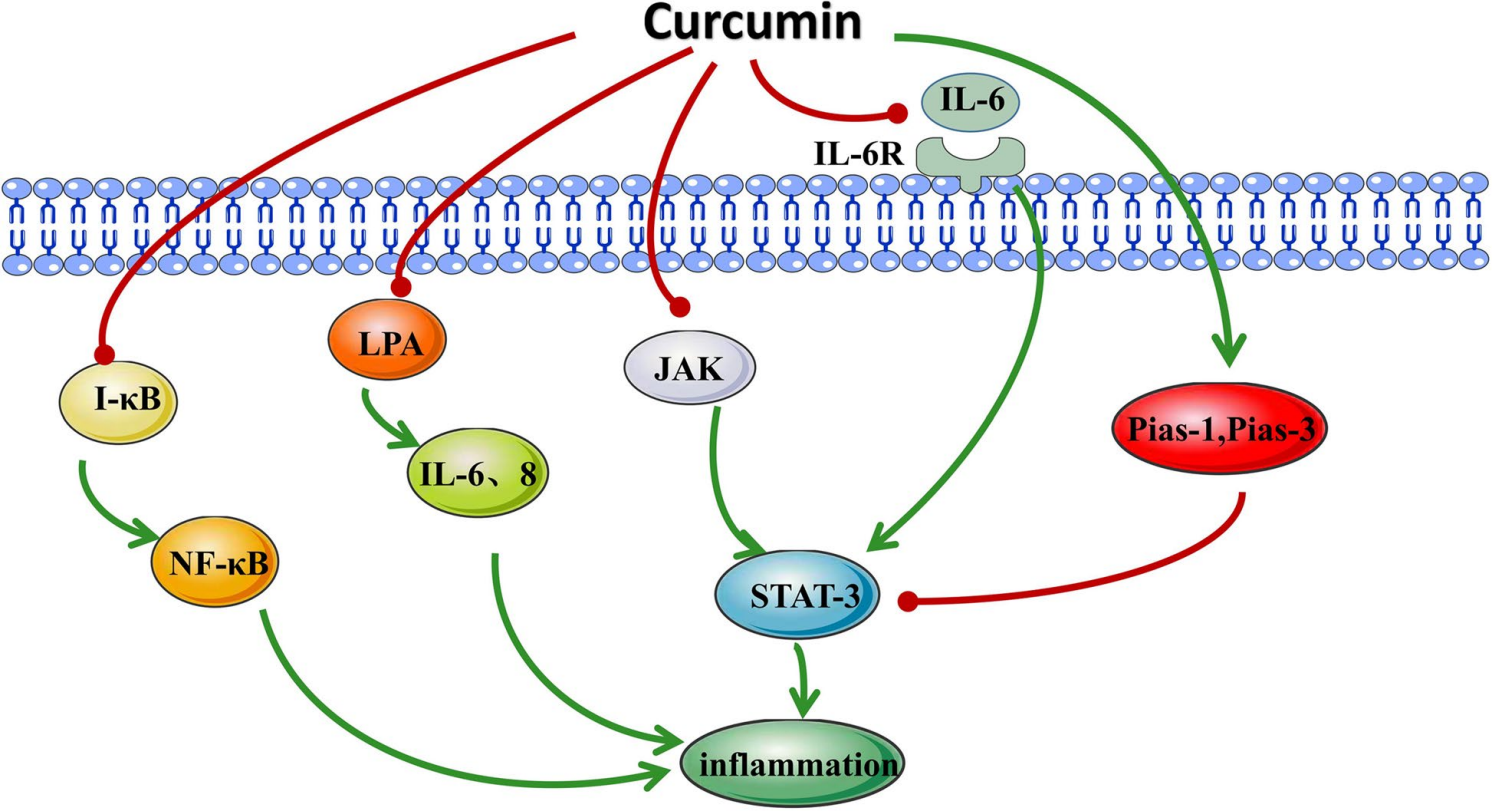
IκB: Inhibitor of κB; NF-κB: nuclear factor kappa B; LPA: Lysophosphatidic acid; IL-6, -8: Interleukin-6, -8; JAK: Janus Kinase; STAT 3: Signal transducer and activator of Transcription 3; Pias-1,-3: Protein inhibitor of activated STAT-1,-3.

Curcumin has obvious anti-inflammatory effects, and its mechanism is mainly through inhibiting the activity and production of inflammatory factors such as NF-κB, TNF-α, IL-6 and IL-8 (Fig. 4). These inflammatory mediators have been shown to implicated in both the initiation and development of cancers. Screening of 20 curcumin analogs showed that curcumin was the most effective analog in inhibiting tumor necrosis factor (TNF)-induced NF-κB activation [68]. NF-κB, a proinflammatory transcription factor, is an important link between inflammation and cancer. Because it is involved in the adjustment of cell adhesion molecules, genes encoding cytokine and cytokine receptors [69]. Phosphorylation of the cytostatic IκB is a key event for NF-κB activation, resulting in the release of p50/p65 heterodimers. In vitro studies confirmed that curcumin is involved in tumor cell growth and angiogenesis through targeting the NF-κB pathway [56]. In vivo studies have also shown that intake of curcumin can suppress NF-κB expression through IκB-mediated mechanisms. IκB binds to the nuclear localization sequence of NF-κB and blocks its function by preventing NF-κB translocation from the cytoplasm to the nucleus [70]. In SKOV3 cells, BDMC can also prevent the activation of the NF-κB pathway, which is related to its decreased phosphorylation levels of p65 (Ser536) and IκB-α (Ser32-36) [54].

It is widely reported that the elevated level of IL-6 is related to the poor prognosis [71] and drug resistance of various cancers [72]. IL-6 can induce the expression of a series of inflammatory factors and inflammatory pathways. Y,W. et al [73] revealed that overexpression of IL-6 in non-IL-6-expressing A2780 cells increases anchorage-independent growth, proliferation, adhesion and invasion. At the same time, depletion of endogenous IL-6 expression in IL-6-overexpressing SKOV-3 cells contribute to tumorigenesis. It has been observed that IL-6 is expressed at high levels in cisplatin-resistant OC cells. IL-6 was inhibited after curcumin treatment of cancer cells, and drug-resistant strains were resensitized to cisplatin. This indicates that one of the mechanisms by which cisplatin and curcumin exert their gian of function is through decreasing the production of IL-6 [74]. The combination of IL-6 and interleukin-6 receptor (IL-6R) can activate the gp130 subunit on the surface of the cell membrane, which in turn activates related conduction pathways such as the IL-6/STAT3 pathway. The IL-6/STAT3 pathway plays a major role in epithelial OC proliferation, anaerobic metabolism, tumor resistance, vascular proliferation, and epithelial-interstitial (EMT) phenotype transformation. Curcumin treatment of OC results in a dose- and time-dependent decrease in IL-6 expression and IL-6-induced constitutive STAT3 phosphorylation, which is related to decreased cell viability and increased caspase-3 cleavage. It was also pointed out that the inhibitory function of curcumin on STAT3 activation is due to the suppression of the expression of its activator JAK [20]. Lysophosphatidic acid (LPA) is a biological lipid and an inflammation-related protein that can stimulate tumor cell invasion and metastasis. LPA stimulates IL-6 and IL-8 secretion, leading to phosphorylation of STAT3. The treatment of cancer cells with curcumin can inhibit this effect of LPA, resulting in the impeded movement of OC cells [75]. The level of STAT3 inhibitors (Pias-1 and Pias-3) could be induced with daily intake of curcumin in the diet [70]. Curcumin reduces fascin expression through JAK/STAT3 pathway inhibition, which interferes with the cellular interactions essential for the metastasis and recurrence of ovarian cancer cells [76]. Similarly, the curcumin analog HO-3867 targets pSTAT3 (Tyr705 and Ser727) in cancer cells and xenograft tumors, thereby inhibiting tumor growth and enhancing the chemotherapy sensitivity of cisplatin-resistant OC [49]. Together, curcumin inhibits inflammation by modulating several signaling pathways and cytokines.

### Effect of curcumin on chemotherapy drugs

Platinum-based and taxane-based chemotherapeutics have a broad-spectrum anti-tumor effect. In the clinical treatment of OC, it was found that the failure of chemotherapy is usually related to the multidrug resistance (MDR). Curcumin overcomes MDR through multiple mechanisms [77], such as reverse membrane transporter-mediated MDR, reverse enzyme system-mediated MDR, and reverse-repair mechanism-mediated MDR. The membrane transporter P-glycoprotein (P-gp) effluxes active drugs to make tumor cells resistant. Paclitaxel is a good substrate for P-gp and cytochrome P450 3A2 (CYP3A2), causing it to have a small inhibitory effect in MDR tumors overexpressing P-gp [68]. Curcumin inhibited the expression of P-gp and CYP3A2 to enhance the bioavailability of paclitaxel and sensitized human ovarian cancer cells expressed P-gp and CYP3A2 to paclitaxel treatment [78–80]. Curcumin administration was shown to inhibit NF-κB activity and down regulate P-gp expression in drug resistant SKOV3(TR) human ovarian adenocarcinoma cells, and significantly reduced its IC_50_ [81].

Further research found that curcumin dose-dependently blocked the efflux of three fluorescent substrates of P-gp (rhodamine 123, calpain-AM, and bodipy-FL-vinblastine), giving rise to significant accumulation of these substrates in the cell, but make no difference on the drug sensitivity of cells that do not overexpress P-gp [82]. Curcumin inhibited OC cell proliferation by suppressing TREK-1 and enhanced the anticancer effect of paclitaxel against ovarian cancer. It has been shown that level of potassium channel TREK-1 is raised in OC cells. Similarly, the growth and proliferation of OC cells can be destroyed by potassium channel inhibitors [83].Also, curcumin inhibits ATPase activity in a concentration-dependent manner to reverse drug resistance [82]. Curcumin has been shown to partially reverse the resistance of SKOV3-TR30 cells to paclitaxel by downregulation of glycogen synthase kinase-3 (GSK-3) [84].

In Cisplatin resistant A2780CP ovarian cancer cells, pretreatment with curcumin in cancer cells not only decreased the expression and transcriptional activity of β-catenin, but also down regulated the expression of Bcl-XL and Mcl-1 pro-survival proteins. Thus demonstrated the effectiveness of a curcumin pre-treatment strategy for chemo-sensitizing cisplatin resistant ovarian cancer cells [85, 86]. Guo et al [87] found that CUGBP- and ETR-3-like family 2 (CELF2)/FAM198B may repress OC progression by inhibiting the MAPK/extracellular-regulated protein kinase (ERK) signaling pathway. Finally, a curcumin-induced increase in CELF2 expression resulted in increased ovarian cancer cell sensitivity to cisplatin. Studies have shown that DNC may make ovarian cancer cells synergistically sensitize to oxaliplatin treatment by regulating proteins in MAPK pathway (including MMP-2, MMP-9, PKC, JNK, P38, PI3K and AKT) [88]. Cisplatin kills cancer cells mainly by establishing intra- and inter-strand DNA crosslinks to prevent DNA replication. The Fanconi Anemia (FA)/BRCA pathway is the DNA damage response pathway required to repair cisplatin crosslinks. And the reactivation of this pathway may be one of the causes of OC acquired cisplatin resistance [89]. Treatment with curcumin would downregulate the DNA damage repair response related to the FA/BRCA pathway, for instance, FANCD2 protein mono-ubiquitination, which is a prerequisite for the DNA damage repair complex to form and relocate to the chromatin at the DNA damage site. In addition to resistance to reverse conversion therapy, curcumin pretreatment can also effectively reduce cancer cell colonies’ formation, greatly reducing the minimum effective dose of radiotherapy [86].

### Effects of curcumin on epigenetic modification

More and more evidences show that epigenetic modification plays a very important role in tumor progression. It can affect gene transcription activity without involving changes in DNA sequence. There are many types of epigenetic modifications which include DNA methylation, microRNA, histone modification and nucleosome localization.

Recent studies have found that curcumin’s anti-cancer properties are related to DNA methylation. The Wnt /β-catenin signaling pathway not only promotes the growth and metastasis of tumor cells, but is also related to the chemotherapy sensitivity and poor prognosis of patients [90]. Secreted frizzled-related proteins (SFRPs) act as negative regulators of the Wnt/β-catenin signaling and play a critical role in the process of carcinogenesis. Hypermethylation of the SFRP5 promoter usually silences it in human cancers [91, 92]. This methylation is associated with cisplatin resistance and overall survival of OC, and the restoration of SFRP5 expression weakens Wnt signaling and inhibits the growth of mouse cancer cells [93]. Curcumin reduces DNA methylation by inhibiting DNA methyltransferase activity and protein expression, improves the expression of SFRP5, and inhibits the Wnt/β-catenin pathway. These results also indicate that curcumin affects the specific downstream effector genes(Cyclin D1 and c-Myc) of Wnt pathway that reduce colony formation [60]. Zhang et al [94] found that curcumin’s ability to weaken extracellular vesicles (EV) to cause drug resistance is also related to epigenetic regulation. Curcumin restore MEG3 expression via demethylation pathway. Moreover, upregulation of MEG3 could reduce EVs mediated transfer of miR-214 in OC cells, so as to decrease drug resistance.

miRNAs are highly conserved non-coding sequences. Their up-regulation or down-regulation influences many processes related to development, proliferation, differentiation and apoptosis. They also take part in tumorigenesis [95] by adjusting a series of signaling pathways related to tumor progression, such as AKT, PTEN, p53, and caspase-3. The anti-tumor impact of curcumin, in part, mediated by miRNA. *miR-9* promotes the proliferation and migration of some malignant tumor cells [96–98], but in OC miR-9 improves the chemotherapy effect through enhancing the sensitivity of cells to DNA damage [99]. Zhao et al [100] found that curcumin up-regulated miR-9 in a dose-dependent manner while inhibiting SKOV3 cell proliferation and activating apoptosis. Conversely, downregulation of miR-9 attenuates curcumin’s inhibitory effect on cell growth, reduces the percentage of apoptosis, further inhibits the Akt/FOXO1 axis, promotes caspase-3 related apoptosis, and exerts its toxic effect on OC cells. Midkine (MK), a heparin-binding growth factor, is obviously higher in multiple cancers in comparison with samples from healthy tissues [101]. The curcumin and dihydroartemisinin (DHA) synergistic treatment of OC significantly reduced the expression and secretion of MK and cooperatively up-regulated the level of miR-124, resulting in decreased viability of SKOV3 cells and increased apoptosis. Furthermore, MK has been confirmed as a direct target of miR-124. miR-124 directly bind with the 3ʹ-untranslated region of MK mRNA, leading to mRNA decay and MK protein expressions reduced, while the miR-124 inhibitor reversed MK expression. The data reveal that curcumin promoted SKOV3 cell apoptosis, at least in part, by the way of miR-124-mediated MK degradation. The combination of DHA with curcumin could remarkably attenuated the tumor growth of xenograft nude mice with no evident signs of toxicity [31]. IRS2 was recognized as a downstream gene of miR-551a in accordance with the bioinformatics databases (Starbase and miRanda) [102]. Overexpression of *miR-551a* often inhibits the malignant progression of tumor cells [103, 104]. IRS2 is known to play an oncogenic character in lots of solid tumors, including OC, and its upregulation is involved in the malignant progression of OC [105]. Recent research results prove that BDMC up-regulates miR-551a to target the reduction of IRS2 expression, inactivates the IRS2/PI3K/AKT axis, and hinders the malignant progression of OC [102].

### Application of curcumin drug-loading system

Although curcumin shows abundant pharmacological activity, its instability, poor water solubility, and low bioavailability still limit its application in clinical treatment [106]. A combinatorial therapy based on lipid and polymer-based nanoparticles has been studied to overcome these limitations. The application of nanotechnology can increase bioavailability of curcumin by increasing the penetration of small intestine, avoiding the degradation in the intestinal condition, prolonging plasma half-life, and improving the efficiency of the therapy [107]. These technologies also show good performance in improving the encapsulation rate, stability, and solubility of curcumin, and have outstanding performance in anti-cancer [108, 109]. Studies also show that curcumin nanoparticles do not rise the concentration of drugs in the plasma, but affect their distribution in the ovary [110].

The stability of curcumin in aqueous media could be adequately enhanced by lipid-based nanoparticles (including liposomes). This is because they can contain hydrophobic curcumin in the membrane and prevent curcumin degradation and precipitation [111]. Compared with free curcumin, embedding curcumin in nanostructured lipid carriers maintains its anti-cancer activity and more effectively reduces cell colony survival rate and improves its anti-tumor activity and bioavailability [112]. Preparation of curcumin-loaded PHEMA nanoparticles (C-PHEMA-NPs) by nanoprecipitation. The results show that G-/G_1_ phase cells are reduced after treatment of SKOV3 cells with C-PHEMA-NPs, which has finer tumor cell regression activity than free curcumin [113]. In addition to lipid-based nanoparticles, diverse polymer-based nanoparticles have been used to enhance curcumin’s curative effects further. Dendrosomal nano-curcumin (DNC), which is curcumin combined with dendrosome as a nano-carrier. Research found that DNC and Cur increased the expression level of MEG3 as a tumor suppressor gene, but decreased HOTAIRE and H19 expression in both SKOV3 and OVCAR3 cells. It is important to note that in all experiments DNC showed greater efficiency than Cur, indicating that using DNC could be more advantages than Cur [114].

It has been reported that curcumin’s targeted-PLGA nanoparticle release is more stable than free curcumin, and antibody binding ability is enhanced [85]. The amount of uptake of these curcumin nanoparticles by cells increased twofold compared to free curcumin, and it also showed higher anti-cancer potential in cell proliferation and clone formation experiments [86]. By using lipid-polymer hybrid nanoparticles (LPHNP), it is possible to achieve controlled release of drugs with high encapsulation efficiency, which combines the structural advantages of polymers and the biomimetic properties of lipids [115]. Compared with the curcumin solution, the cytotoxicity and chemical sensitivity to cisplatin of curcumin-loaded LPHNPs are increased [116].

Furthermore, abundant studies have shown that a curcumin and chemotherapeutic drug co-delivery system is able to improve the anti-tumor efficacy while do not increase adverse reactions [117]. Co-loading curcumin and paclitaxel into mixed micelles made of PEG-PE/vitamin E has significant advantages both in vivo and in vitro, especially when treating drug-resistant tumors [118]. Compared with the free drug, PLGA phospholipid-PEG nanoparticles loaded with paclitaxel and curcumin improve the drug’s solubility and stability, slowing down the drug release and significantly reducing the P-gp content of drug-resistant cell lines to overcome MDR, thus improving the efficacy of chemotherapy drugs [79]. This targeted nanoparticle has broad application prospects and shows good anti-cancer effects in nude mice. Its special structure enhances the anti-tumor efficacy on the one hand, and decreases the adverse side effects of the drug on the other hand [119]. These results indicate that nanotechnology is a promising strategy to enhance the pharmacokinetics of curcumin.

Some drug-loading systems containing special ingredients have been studied, such as PEG-PE mixed micelles targeted by transferrin (TF). Iron is essential for cellular DNA synthesis. Not surprisingly, transferrin (TF) receptors are increased in OC cells. Consequently, TF-targeted combined micelles with paclitaxel and curcumin heightened the inhibitory function on OC cells via inhibition of NF-κB and AKT [120]. A few studies have revealed the cytotoxicity of curcumin-coated silver nanoparticles (cAgNPs) on cancer cells. Silver binds to protective SH groups at the surface of the cell membrane, leading to morphological changes in the plasma membrane’s permeability. Furthermore, silver affects the respiratory chain and causes cell death. The higher metabolic rate of cancer cells makes them more sensitive to silver nanoparticles. A mixture of cisplatin and cAgNPs activates p53 in A2780 CR cells, showing higher apoptotic activity [121].

### Additional molecular studies

Malignant epithelial OC spheroids and high levels of cancer stem cell (CSC) marker, such as aldehyde dehydrogenase 1 family member A1(ALDH1A1), are frequently discovered in malignant ascites of patients with extensive peritoneal metastasis of OC. Curcumin suppresses the formation of EOC spheres in a concentration-dependent manner while reducing the expression of ALDH1A1. Curcumin also has the ability to weaken the adhesion and invasion of EOC spheres to the extracellular matrix and mesothelial monolayer, thereby inhibiting metastasis [122]. A curcumin analogue GO-Y030 depletes cancer stem cells by inhibiting the interaction between the HSP70/HSP40 complex and its substrates [123].

Curcumin induces the overexpression of folate receptor-α, resulting in enhanced uptake of paclitaxel in tumor cells, cytotoxicity, and anti-cancer effects [124]. The occurrence and development of OC are strongly relevant to oxidative stress (OS). In various animal models, curcumin also inhibits oxidative damage [70, 125, 126]. It can reduce the ovarian ischemia/reperfusion(I/R) injury by decreasing OS markers [127]. As a recognized antioxidant, curcumin can inhibit LPA-dependent NF-κB activity and the proliferation of SKOV3 cells [126]. Glutathione (GSH) is an important antioxidant and free radical scavenger in the body. The level of GSH in CR without curcumin treatment is obviously higher than that of cisplatin-sensitive cells (CS). The GSH level increased in treated CR and CS cells, indicating that cisplatin resistance in OC is related to increased intracellular GSH content. However, the antiproliferative effects of curcumin on CR and CS are not significantly different, indicating that curcumin induces apoptosis through a GSH-independent pathway [44].

## Conclusion

In summary, curcumin and its analogs strongly inhibit OC. The molecular mechanisms involve apoptosis, cell cycle progression, cell autophagy, tumor invasion, metastasis ability, enzyme activity in vivo, and antioxidant effects, and can reverse the multidrug resistance of tumor cells. A wide variety of signaling pathways, including PI3K/AKT, NF-κB, P53, and AKT/mTOR/p70S6K, are participated in the regulation of OC cells biological behavior, and non-coding RNAs such as miRNA also play a role, which shows that curcumin’s anti-tumor properties are based on multiple pathways, targets, and systems.

Clinical experiments have revealed some promising outcomes. Around 210 clinical trials on curcumin application have been documented. Several clinical trials have shown that curcumin has beneficial effects on serum markers of inflammation, weight loss and glucose and lipid metabolism. Although clinical trials have shown that healthy subjects and patients with cancer tolerated oral curcumin well with few side effects, rapid drug metabolism, poor solubility, and low bioavailability limit their clinical application. Therefore, more effective curcumin analogs and various nanocarriers have been developed. Many clinical trials have shown that nanocurcumin is useful in treating familial adenomatous polyposis, rheumatoid arthritis, chronic kidney disease, metabolic syndrome patients and malignancies. It can be concluded that the therapeutic potentials of curcumin may be enhanced by improving its bioavailability and solubility. With the continuous deepening of research on the molecular mechanism of curcumin’s anti-tumor effect, its unique role in anti-tumor and wide application prospects will further attract attention, and curcumin is expected to become an attractive target for the development of anti-cancer drugs.

## Data Availability

This manuscript is a review paper. So, “Not applicable”.
